# Phylogenetic relationship and characterization of the complete mitochondrial genome of *Milionia basalis* (Lepidoptera: Geometridae)

**DOI:** 10.1080/23802359.2019.1692732

**Published:** 2019-11-20

**Authors:** Yimin Du, Xiang Song, Zhanjun Lu

**Affiliations:** a School of Life Sciences, Gannan Normal University, Ganzhou, China;; b National Navel Orange Engineering and Technology Research Center, Ganzhou, China

**Keywords:** Geometridae, mitochondrial genome, *Milionia basalis*, phylogenetic analysis

## Abstract

Larvae of the geometrid moth *Milionia basalis* feed exclusively on the podocarp tree, *Podocarpus macrophyllus*. In this study, we sequenced and analyzed the complete mitochondrial genome (mitogenome) of *M. basalis*. This mitogenome was 15,901 bp long and encoded 13 protein-coding genes (PCGs), 22 transfer RNA genes (tRNAs), and two ribosomal RNA unit genes (rRNAs). The whole mitogenome exhibited heavy AT nucleotide bias (82.2%). Gene order was conserved and identical to most other previously sequenced Geometridae. Most PCGs of *M. basalis* had the conventional start codons ATN, with the exception of *nad1* (TTG) and *cox1* (CGA). Except for four genes (*cox1*, *cox2*, *nad5*, and *nad4*) end with the incomplete stop codon T––, all other PCGs terminated with the stop codon TAA. Phylogenetic analysis showed that *M. basalis* got together with *Apocheima cinerarius*, *Jankowskia athlete*, and four *Biston* species (*B. panterinaria*, *B. perclara*, *B. suppressaria*, and *B. thibetaria*).

The medium-sized geometrid moth *Milionia basalis* Druce (Lepidoptera: Geometridae) has orange-banded dark blue wings in the adult stage and is found in some parts of subtropical Asia (Shintani et al. 2018). The larvae of this species feed exclusively on *Podocarpus macrophyllus* (Podocarpaceae) which is often used as building material or garden tree in some areas (Yasui et al. 2005).

Specimens of *M. basalis* were collected from Jinggangshan City, Jiangxi Province, China (26°34′N, 114°06′E, July 2019) and were stored in Entomological Museum of Gannan Normal University (Accession number GNU-MB055). After morphological identification, total genomic DNA was extracted from tissues using DNeasy DNA Extraction kit (Qiagen, Hilden, Germany). The mitogenome sequence of *M. basalis* was generated using Illumina HiSeq 2500 Sequencing System (Illumina, San Diego, CA). In total, 5.2 G raw reads were obtained, quality-trimmed and assembled using MITObim v 1.7 (Hahn et al. 2013). By comparison with the homologous sequences of other Geometridae species from GenBank, the mitogenome of *M. basalis* was annotated using software GENEIOUS R8 (Biomatters Ltd., Auckland, New Zealand).

The complete mitogenome of *M. basalis* is 15,901 bp (GenBank accession, MN495623). It contains 13 protein-coding genes (PCGs), 22 tRNA genes, two rRNA genes, and one non-coding AT-rich region. The nucleotide composition of the mitogenome was biased toward A and T, with 82.2% of A + T content (A 41.6%, T 40.6%, C 10.5%, G 7.3%). Gene order was conserved and identical to most other previously sequenced Geometridae (Liu et al. 2014; Chen et al. 2016; Cheng et al. 2017; Sun et al. 2017; Li et al. 2018). Four PCGs (*nad4*, *nad4l*, *nad5*, and *nad1*) were encoded by the minority strand (N-strand) while the other nine were located on the majority strand (J-strand). Most PCGs of *M. basalis* had the conventional start codons ATN (five ATG, five ATA, and one ATT), with the exception of *nad1* (TTG) and *cox1* (CGA). Except for four genes (*cox1*, *cox2*, *nad5*, and *nad4*) end with the incomplete stop codon T––, all other PCGs terminated with the stop codon TAA. Two rRNA genes (*rrnL* and *rrnS*) located at *trnL1*/*trnV* and *trnV*/control region, respectively. The lengths of *rrnL* and *rrnS* in *M. basalis* were 1670 and 789 bp, with the AT contents of 86.9% and 86.6%, respectively. The 22 tRNA genes vary from 65 bp (*trnE*, *trnG*, and *trnT*) to 71 bp (*trnK*).

Phylogenetic tree was constructed using the maximum-likelihood method through raxmlGUI 1.5 (Silvestro and Michalak 2012) based on 13 mitochondrial protein-coding genes sequences. Results showed that the new sequenced species *M. basalis* got together with *Apocheima cinerarius*, *Jankowskia athlete* and four *Biston* species (*B. panterinaria*, *B. perclara*, *B. suppressaria*, and *B. thibetaria*), indicating the close relationship of these four genus (Figure 1). In conclusion, the mitogenome of *M. basalis* is sequenced in this study and can provide essential DNA molecular data for further phylogenetic and evolutionary analysis of Geometridae.

**Figure 1.**
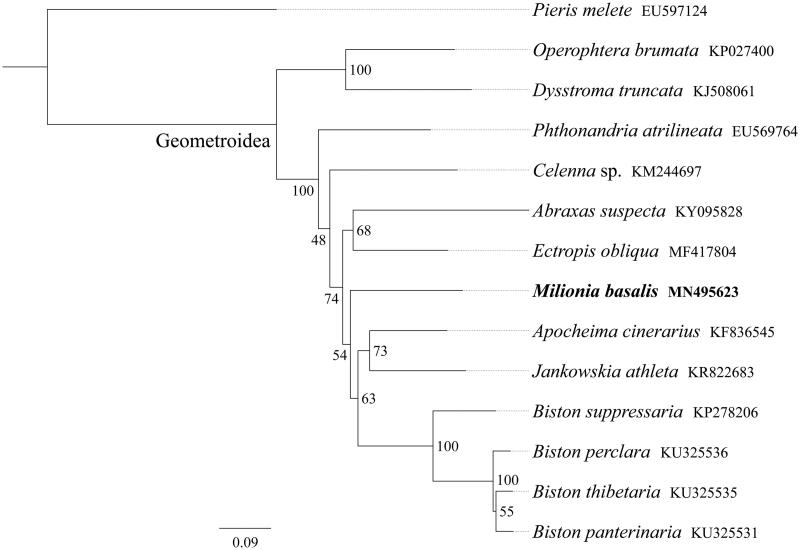
Phylogenetic relationships based on the 13 mitochondrial protein-coding genes sequences inferred from RaxML. Numbers on branches are Bootstrap support values (BS).
